# Long-term evaluation of mucosal and systemic immunity and protection conferred by different polio booster vaccines

**DOI:** 10.1016/j.vaccine.2016.12.061

**Published:** 2017-09-25

**Authors:** Yuhong Xiao, Henry Daniell

**Affiliations:** Department of Biochemistry, School of Dental Medicine, University of Pennsylvania, Philadelphia, PA 19104, USA

**Keywords:** Poliovirus, Polio viral protein 1 (VP1), Bioencapsulated plant cells, Oral delivery

## Abstract

Oral polio vaccine (OPV) and Inactivated Polio Vaccine (IPV) have distinct advantages and limitations. IPV does not provide mucosal immunity and introduction of IPV to mitigate consequences of circulating vaccine-derived polio virus from OPV has very limited effect on transmission and OPV campaigns are essential for interrupting wild polio virus transmission, even in developed countries with a high coverage of IPV and protected sewer systems. The problem is magnified in many countries with limited resources. Requirement of refrigeration for storage and transportation for both IPV and OPV is also a major challenge in developing countries. Therefore, we present here long-term studies on comparison of a plant-based booster vaccine, which is free of virus and cold chain with IPV boosters and provide data on mucosal and systemic immunity and protection conferred by neutralizing antibodies.

Mice were primed subcutaneously with IPV and boosted orally with lyophilized plant cells containing 1 μg or 25 μg polio viral protein 1 (VP1), once a month for three months or a single booster one year after the first prime. Our results show that VP1-IgG1 titers in single or double dose IPV dropped to background levels after one year of immunization. This decrease correlated with >50% reduction in seropositivity in double dose and <10% seropositivity in single dose IPV against serotype 1. Single dose IPV offered no or minimal protection against serotype 1 and 2 but conferred protection against serotype 3. VP1-IgA titers were negligible in IPV single or double dose vaccinated mice. VP1 antigen with two plant-derived adjuvants induced significantly high level and long lasting VP1-IgG1, IgA and neutralizing antibody titers (average 4.3–6.8 log2 titers). Plant boosters with VP1 and plant derived adjuvants maintained the same level titers from 29 to 400 days and conferred the same level of protection against all three serotypes throughout the duration of this study. Even during period, when no plant booster was given (∼260 days), VP1-IgG1 titers were maintained at high levels. Lyophilized plant cells expressing VP1 can be stored without losing efficacy, eliminating cold chain. Virus-free, cold-chain free vaccine is ready for further clinical development.

## 1 Introduction

Poliovirus, the causative agent of poliomyelitis, is a human enterovirus with an RNA genome (7.5 kbp) and a capsid protein. Because of its smaller size (30 nm diameter) and simple structure, it has been studied extensively. Poliovirus enters human cells by binding to CD15, an immunoglobulin like receptor and endocytosis [1], [2]. Because poliovirus is a positive stranded RNA virus, upon entry into human cells, it is readily translated. Poliovirus hijacks the cell by producing a protease that destroys the cap binding proteins; because translation of poliovirus mRNAs is cap-independent, host cell translational machinery becomes totally dedicated for production of viral proteins. Inhibition of host translational system in favor of virus specific protein synthesis results in production of a single long protein, which is cleaved into ten viral proteins by internal proteases.

Poliovirus enters human body through the fecal-oral route and the virus is shed in the feces of infected individuals, posing a major problem in eradication of this disease. Even in countries where public sewer system is well protected, silent polio outbreaks have been detected. Upon careful environmental monitoring a silent polio outbreak was recently reported in Israel [3], [4] but most countries including the United States such monitoring is not done. In a large majority of infected patients poliovirus is detected in the bloodstream and such infections are asymptomatic. However, in some cases the virus spreads, replicates leading to minor symptoms including fever, headache and sore throat. Paralytic poliomyelitis occurs when poliovirus enters the central nervous system crossing the blood brain barrier [5] and replicates within the spinal cord or brain, causing destruction of motor neuron leading to temporary or permanent paralysis. There are three known serotypes of poliovirus (type 1 – Mahoney, type 2 – Lansing, type 3 – Leon), each with a slightly different capsid protein and all three forms are highly infectious. The outer surface of capsid contains viral protein 1 (VP1), which is the same protein in all poliovirus serotypes and is therefore an ideal antigen for development of vaccines.

Two different polio vaccines were developed sixty years ago. The oral polio vaccine (OPV) contains a mixture of three different polioviruses with mutations to decrease their virulence. There are 57 nucleotide substitutions in the Sabin 1, two in Sabin 2 and ten in Sabin 3 stains that distinguish attenuated strains from virulent strains and reduce ability of poliovirus to translate in the host cell. Attenuated strains escape the acid and enzymes in the human gut and replicate efficiently but are unable to replicate in the central nervous system. OPV eliminated the need for sterile syringes required by IPV and generated mucosal immunity, protecting the primary site of poliovirus entry making this an ideal vaccine for global regions where this virus is endemic and reinfection is more common. Unfortunately, genetic stability of Sabin strains has been a major problem. Vaccine associated paralytic poliomyelitis among recipients of OPV was observed in several outbreak areas in the USA [6], [7], Haiti [8], Dominican Republic [8], India [9], Phillipines [10], African continent [11], [12] and many other global regions. In order to control polio outbreaks, several doses of OPV (as many as 13 doses) were administered [9] but resulted in several cases of vaccine induced polio [13]. In order to address these concerns, WHO Strategic Advisory Group of Experts (SAGE) recommended withdrawal of OPV 2 and the Global Polio Eradication Initiative is facilitating the switching of bivalent OPV from trivalent OPV in summer 2016 in many countries around the globe.

The Inactivated Polio Vaccine (IPV) is safe but less efficient in inducing mucosal immunity that is needed to prevent reinfection. Moreover, IPV required multiple boosters to maintain immunogenicity against polio virus infection. It is also not affordable in many developing countries. The high cost and limited supply of IPV has led SAGE to propose that one dose of IPV is adequate to prime population immunity. One dose of IPV has been adapted into routine immunization systems to boost immunity against poliovirus types 1 and 3 and provide a baseline of immunity against type 2 in case of an outbreak of type 2 vaccine derived poliovirus. It is indeed a major challenge to supply IPV globally. However, a diluted (or fractional) dose IPV can overcome this problem. Traditionally, full dose IPV is delivered through an intramuscular injection. However, when delivered subcutaneously, only 1/5 of a full dose IPV can generate almost as much immunity as one full dose delivered into the muscle; and two fractional doses generates higher immunity than one full dose [14]. These two alternative delivery routes could reduce the cost of IPV immunization and enable wider use of the limited supply of IPV. Adding to previous studies, a new field study in Sri Lanka provided more evidence that using fractional dose IPV is as effective as using a full dose in OPV primed populations to boost mucosal immunity [15].

The Global Polio Eradication Initiative (GPEI) was established in 1988 as a public-private partnership led by national governments and spearheaded by the World Health Organization (WHO), Rotary International, the US Center for Disease Control, the United Nations Children’s Fund (UNICEF) and with substantial support from the Bill & Melinda Gates Foundation [16]. GPEI brought together under one umbrella recent scientific advances on poliovirus and kept track of polio around the world. Afghanistan, Nigeria and Pakistan are-still listed as endemic areas globally for poliovirus. GPEI is working hard to strengthen global surveillance and immunization systems. The final goal of polio eradication by GPEI is “the endgame strategic plan” to detect and stop all wild-type poliovirus transmissions, including withdrawal of the use of OPV2 in the oral vaccine. The GPEI is still exploring additional delivery methods to overcome potential operational challenges, such as adaptors and needle-free devices to make it easier to deliver the vaccine, especially for children.

While IPV was effective in saving lives, several recent studies show that lack of mucosal immunity is a major challenge in eradication of polio and prevention of transmission. Polio eradication efforts are hampered by reintroduction of virus in polio free countries. Recent silent polio outbreak observed in Israel, which has used IPV for many decades, is one such example. Environmental surveillance in the absence of paralytic cases in 2013 revealed the presence of wild poliovirus in sewage samples in the South, Central and northern parts of Israel [4]. Open sewer system in many developing countries renders IPV unsuitable for polio eradication and environmental surveillance is not meaningful. Therefore, finding an alternative booster vaccine to stimulate both systemic and mucosal immune response after priming with IPV is indeed necessary.

From discussions above, the advantages and limitations of both OPV and IPV are quite evident. While IPV has not resulted in vaccine derived poliomyelitis, it does not provide mucosal immunity and therefore is not suitable for polio eradication or prevention of transmission. Indeed, in depth studies show that introduction of IPV to mitigate consequences of circulating vaccine-derived polio virus will have very limited effect on transmission and OPV campaigns are essential for interrupting wild polio virus transmission, even in a developed country with a high coverage of IPV and protected sewer system [4]. These conditions are not realistic to achieve in many countries with poor resources. Furthermore, switching from trivalent OPV to bivalent OPV will reduce protection against type 2 poliovirus and could lead to reintroduction of this poliovirus [4]. Requirement of refrigeration for storage and transportation for both IPV and OPV is also a major challenge in developing countries. Therefore, we have recently developed a plant-based booster vaccine which is free of virus and cold chain [17]. In this study, we compare long-term efficacy of this booster vaccine with IPV prime/boost, evaluate mucosal and systemic immunity and protection conferred by both types of vaccines.

## 2 Materials and methods

### 2.1 Plant-made protein and vaccine formulation

As previously described [17], lyophilized plant cells containing 1 μg or 25 μg of viral protein 1 (VP1) and plant-made adjuvants, saponion and/or squalene, were used for oral boosting. Briefly, an oil/water (O/W) emulsion was made by mixing the primary oil emulsion (squalene and Span 80) with the aqueous phase (saponin and lyophilized VP1) and adjusting the total volume to 200 μl per mouse with PBS.

### 2.2 Mice and immunization study

**Fig. 1:**
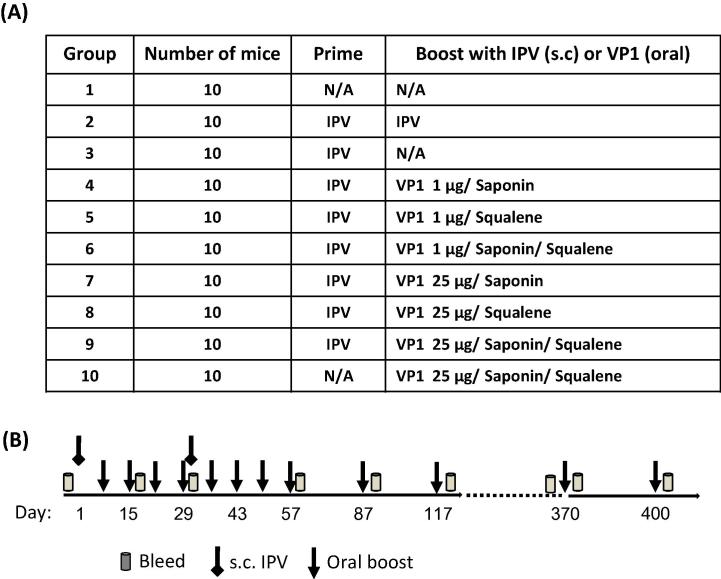
Design of long-term in vivo polio vaccine study. (A) Female CD-1 mice were randomly divided into 10 groups. Lyophilized plant cells expressing 1 μg or 25 μg of viral protein 1 (VP1) and plant-derived adjuvants (Saponin and/or Squalene) were used in this study. (B) All groups of mice except group 1 and group 10 were subcutaneously primed with IPV on day 0, and Group 2 mice were boosted with the same IPV 30 days after priming. Group 4–10 were orally boosted with lyophilized plant cells expressing VP1 once a week for 8 weeks, followed by once a month for three months. One year after priming, group 4–10 mice were boosted once a month for two times.

Six-week-old female CD-1 mice (Charles River Laboratories, Wilmington, MA, USA) were housed in micro-isolator cages. Totally there are ten groups of mice vaccinated with various formulations (group 3–10) (Fig.1A and B). Group 1 was untreated. Mice were subcutaneously (s.c) primed and boosted with IPV (Groups 2) or prime only (group 3). Mice were orally boosted with either 1 μg VP1 from expressing leaves (group 4–6) or 25 μg (group 7–10), once a week for 8 consecutive weeks, followed by oral boosting once a month for three months. Six month after last boosting, mice in groups 4–10 were boosted with VP1 with one or two adjuvants once a month for two times. Blood was collected one day prior to priming and 10 days after each boost.

### 2.3 Determination of antibody responses by ELISA, poliovirus neutralization titers and seropositivity rate

#### 2.3.1 Antibody titers assay by ELISA

Mice were bled 1 day prior to priming and 10 days after boosting. As previously described [17], all collected serum samples were inactivated at 56 °C for 30 min to inhibit complement activity. Serum VP1-specific IgG1 and IgA were assayed by direct ELISA as previously reported [18], [19]. Briefly, 96-well ELISA plates were pre-coated with 10 μg/ml purified VP1 protein, and incubated with twofold dilutions of heat-inactivated individual serum samples starting dilutions at 1:400 for VP1-IgG1 and 1:40 for VP1-IgA, overnight at 4 °C and probed with anti-mouse secondary antibodies. The absorbance and antibody titers were determined as previously described [17], [20]. All serum samples were tested in triplicate.

#### 2.3.2 Poliovirus Sabin 1, 2, 3 neutralization assay

Individual serum samples were collected 10 days after boosting for neutralization assays performed at Centers for Disease Control and Prevention (CDC) as previously described [14], [21], [22], [23]. The reciprocal titer at which no virus neutralization was detected (negative) was recorded as the log_2_ (titer) of 2.5, whereas a log_2_ titer of ⩾3 was considered protective. Individual titers for each mouse are plotted and the bar represents mean neutralizing titer ± SEM.

### 2.4 Statistical analysis

Data are reported for individual mice and groupings and mean ± SEM is given for each group. Analyses for statistically significant differences in antibody titers between groups were performed using Student’s *t*-test (GraphPad Prism version 6) and *P* values <0.05 were considered significant.

## 3 Results

Our prior study [17] has shown that short-term oral boosting with plant derived VP1 expressed in leaves plus adjuvants elicited highly specific mucosal and systemic antibody immune response as well as neutralizing titers. In this study we continued to orally boost these mice once a month, with a six month gap without any boosting, followed by a single boost one year after the date of first priming (Fig.1B). The purpose of this long-term vaccination study is to evaluate longevity of the antibody titers and ability to boost waning immunity to recall immune response memory. We compared long-term efficacy of plant boosters with IPV prime/boost in terms of maintaining functional immunity response (Fig.1A).

### 3.1 VP1 plant formulation provided long-lasting high antibody titers

**Fig. 2:**
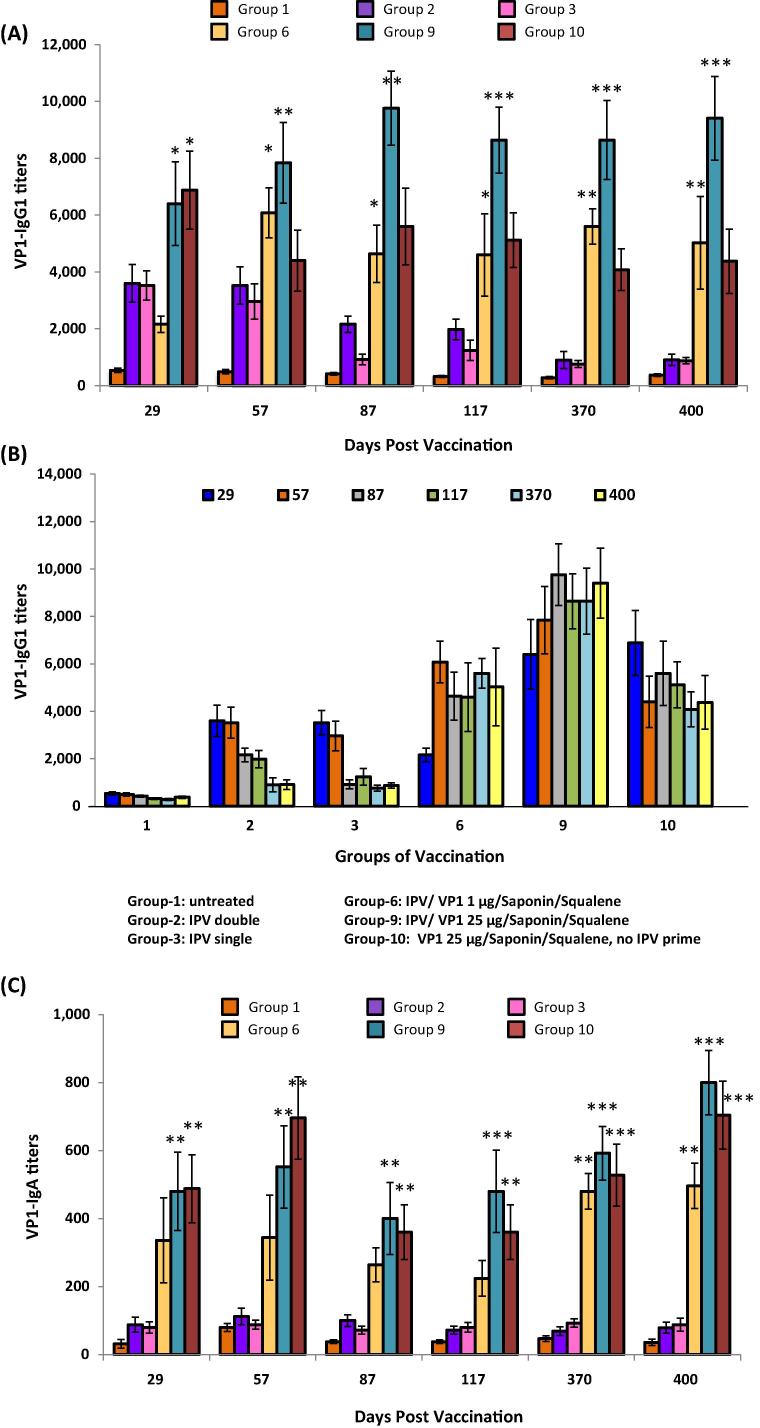

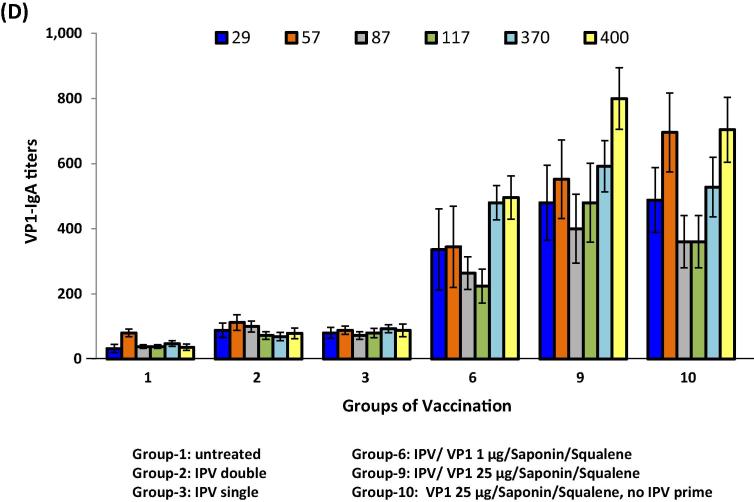
Kinetic antibody response of groups of mice after oral and/or subcutaneous vaccination. Serum VP1-IgG1 (A and B) and VP1-IgA (C and D) antibody titers were assayed by direct ELISA in 96 well plates pre-coated with purified VP1 protein (10 μg/ml). Antibody titers from six groups of mice are shown: untreated group, single or two doses of IPV, priming with IPV and oral boosting with 1 μg or 25 μg plant VP1 protein with two adjuvants (saponin/squalene), and oral boosting with VP1 formulation but without IPV priming at different time points: 29, 57, 87, 117, 360, 370 and 400 days after priming. Statistical analysis (by Student’s *t*-test) (GraphPad Prism version 6) are noted with ^*^P < 0.05, ^**^P < 0.01, ^***^P < 0.001.

Mice boosted with plant cells containing 25 μg of cholera non-toxic B subunit fused VP1 protein (CTB-VP1) with both adjuvants (group 9) had highest mean anti-VP1 IgG1 antibody titers at 87th, 117th, 370th and 400th days (the range of mean titers: from 8640 to 9760). The additional boosters at six months after last boosting did not increase IgG1 titers, confirming the IgG1 antibody titers has been elicited and maintained at high levels after initial short-term vaccination. The same pattern was observed using 1 μg of VP1 plant material but with much lower antibody titers (mean titers from 4640 to 5600 for day 87, 117, 370 and day 400) (Fig.2A and B). Therefore, the amount of protein in the vaccine formulation is critical for producing specific antibodies for immune responses. However, VP1-IgG1 titers in mice vaccinated with one or two doses of IPV steadily declined from 3520 to 876 and 3600 to 911 after the first month and remained low.

To investigate mucosal immune responses, we measured serum IgA since our previous work had already shown higher specific VP1-IgA titers in fecal extracts [17]. Results showed that serum VP1-IgA titers increased in the first month after oral boosting and remained at relatively high level for a long period from 117 days (480) through 370 days (592) without any boosting. Serum VP1-IgA titers were negligible in single or double dose IPV, potentially confirming limitations of long-term protection after systemic vaccine delivery in IPV vaccination (Fig. 2 C and D). In contrast, serum VP1-IgA steadily increased to 496 and 800 on day 400 from 264 and 400 on day 87 with either 1 μg or 25 μg of VP1. These results show that oral boosting with plant cells expressing CTB-VP1 can elicit an entire year of prolonged, high-level mucosal and systemic immune responses but that single or double dose IPV resulted in lower IgG1 and negligible IgA titers.

### 3.2 VP1 plant formulation induced long-lasting, high titers of poliovirus-neutralizing antibodies

All individual blood samples were tested in triplicate in a double-blind manner at CDC. Prior work has shown that after IPV priming, mice orally boosted with either 1 μg or 25 μg VP1 formulations, as well as one or two doses of IPV, induced different levels of neutralizing antibody titers against all three Sabin strain serotypes [17]. We used this as the basis to study kinetic changes of neutralizing antibody titers in different groups during the year, especially after one year. Second, we examined if neutralizing titers could be maintained in groups of mice orally boosted with high-dose VP1.

**Fig. 3:**
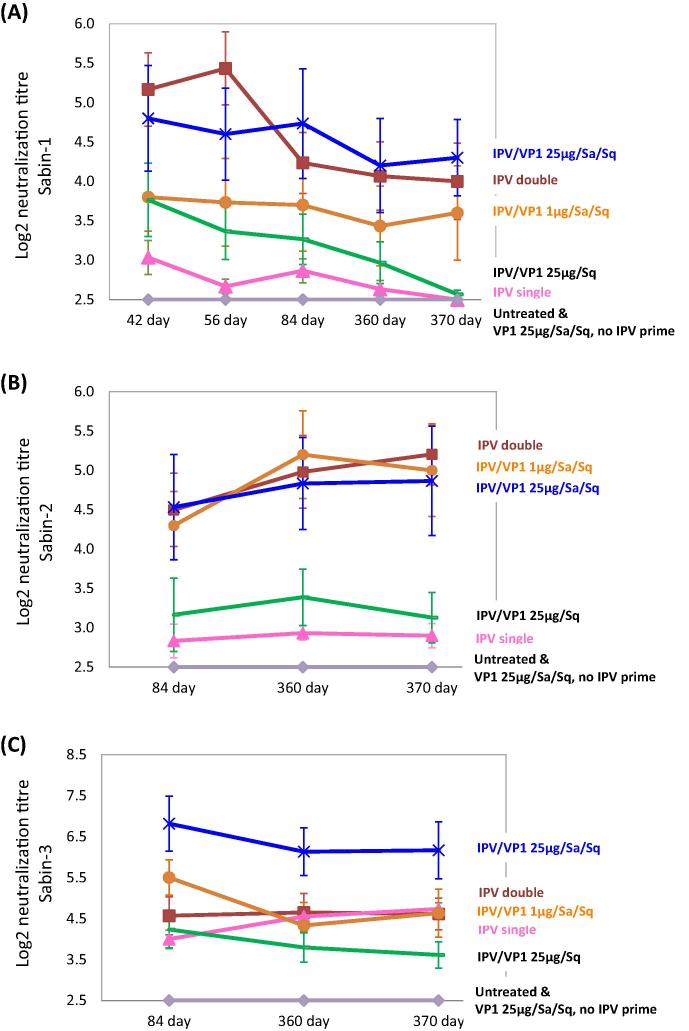
Determination of poliovirus neutralizing antibodies against all three Sabin strains after subcutaneous IPV or oral VP1 boosting. (A) Sabin 1 at 42, 56, 84, 360 and 370 days after prime, (B) Sabin 2 and (C) Sabin 3 at 84, 360 and 370 days after prime. Data are from seven groups: CTB-VP1 antigens (1 μg or 25 μg) adjuvanted with saponin and squalene (group 6, 9 and 10), or squalene only (group 8), two doses of IPV (group 2) or single IPV dose (group 3); and untreated mice (group 1). Each dot represents the mean neutralizing titer ± SEM. The serum dilution of a reciprocal titer at which no virus neutralization was detected was recorded as the log_2_ (titer) of 2.5. The Student’s *t*-test (GraphPad Prism version 6) was used for statistical analysis.

As shown in Fig. 3, oral boosting with plant cells containing 25 μg VP1 induced highest levels of Sabin 1-, 2- and 3-neutralizing antibodies, and they remained at high level during the year. By contrast, mice that received double dose IPV decreased for Sabin 1 from 5.1 log_2_ on day 42 to 3.8 log_2_ on day 370, whereas VP1 boosters with plant cells (25 ug) maintained neutralizing titers at 4.8 log_2_ and 4.4 log_2_ levels.

IPV neutralization with single IPV was lowest in all tested samples at all time points for Sabin 1 and Sabin 2 serotype, but was similar to double IPV vaccination in Sabin 3 serotype. However, plant boosters without IPV priming did not generate neutralizing antibodies, confirming that boosting with the subunit vaccine alone could not induce any protective neutralizing antibodies. Plant boosters with Squalene alone was lower either for Sabin 2 and Sabin 3 or steadily decreased from 3.7 log_2_ on day 42 to 2.5 log_2_ on day 370 for Sabin 1 serotype. IPV double dose resulted in Sabin 2 neutralization titers that steadily increased or were maintained in the range of 4.5 to 5.2 log_2._ Sabin 3 neutralization titers were highest in 25 μg plant boosters in the 6.1–6.9 log_2_ range and were maintained steadily from 84 days through 370 days. IPV double and single dose maintained neutralizing titers in the lower range (4.5 to 4.6 log_2_ and 3.9 to 4.7 log_2_) from 84 days to 370 days.

**Fig. 4:**
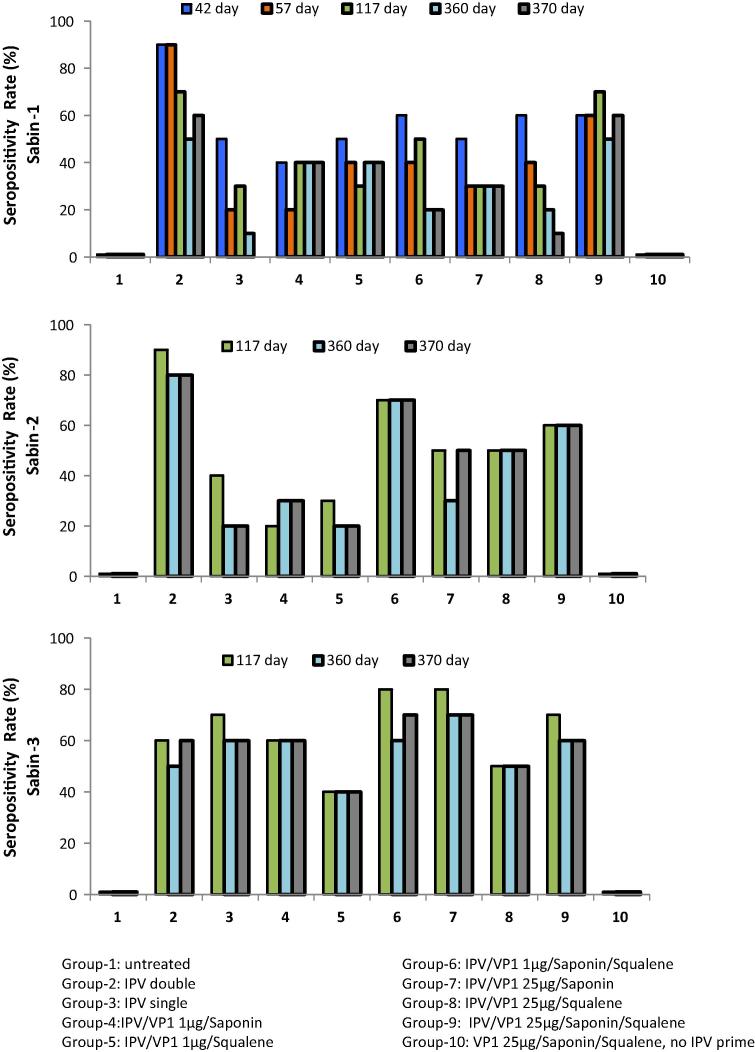
Determination of seropositivity rate of Sabin 1, 2 and 3 neutralizing titers after subcutaneous IPV or oral VP1 boosting. The seropositivity rate of poliovirus-neutralizing antibodies are determined by the number of mice with seroprevalence (neutralizing antibody log_2_(titer) ⩾3) with the total number of mice in each group boosted with 1 μg or 25 μg CTB-VP1 (Groups 4–10), or, IPV two doses (Group 2) at day 1 and day 30 or IPV single dose (Group 3). The kinetic change of seropositivity rate of neutralizing titers against Sabin strains 1, 2 and 3 at 42, 57, 117, 360 and 370 days after priming are shown.

To determine the seropositivity rate of poliovirus-neutralizing antibodies, the number of mice with seroprevalence (neutralizing antibody log_2_ (titer)⩾3) was compared with the total number of mice in each group (Fig. 4). Our prior work has shown that mice given two doses of IPV or primed with IPV then orally boosted with codon-optimized VP1 antigen plus saponin and squalene adjuvants showed the highest seropositivity for poliovirus Sabin 1-, 2- and 3-serotypes [17]. In the current experiment, we determined if the long-term seropositivity rate changed after vaccination.

Maximal variation in seropositivity was observed against Sabin 1. In single dose IPV it decreased from 50% on day 42 to 10% on day 370. Likewise in double dose IPV seropositivity decreased from day 42 (90%) to 60% on day 370. Similar steady decreases were observed in Squalene VP1 boosted group (25 μg) from 60% on day 42 to 10% on day 370. In contrast, in 25 μg plant boosters with both adjuvants seropositivity had no decrease and were maintained at 60% from day 42 to day 370. There was no difference in seropositivity of double dose IPV, either with 1 μg or 25 μg plant boosters against Sabin 2 or Sabin 3 serotype.

VP1-IgG1 titers correlated directly with Sabin 1 seropositivity in both single and double dose IPV, when Squalene alone was used as an adjuvant or with both adjuvants in plant boosters. However, Sabin 2 or 3 serotype seropositivity did not correlate with IgG1 titers. Without IPV priming, there was no correlation between plant cell booster and antibody titers.

Overall, these results showed that oral boosting with plant cells expressing 25 μg VP1 after IPV priming (group 9) generated highest neutralizing antibody titers and seropositivity rates, and they remained high over the entire period (more than one year). A single dose of IPV (recommended by WHO) results in very low neutralizing titers and seropositivity against all three polio serotypes, and both decrease during the first year after immunization.

## 4 Discussion

In order to prevent cases of Vaccine Derived Poliovirus (VDPV), OPV 2 was withdrawn from use globally earlier this year and is currently replaced by including a single dose of bivalent IPV. This action raises a number of important questions to be addressed. What is the level of protection against strain 2 conferred by one or two doses of IPV? What is the duration of such protection? Would IPV prevent transmission of wild type or VDPV? Environmental surveillance has also identified WPV in countries with prolonged and exclusive use of IPV. If withdrawal of OPV2 reduces protection against type 2 polioviruses, what are the next steps required to prevent a new epidemic or fully eradicate polio? Therefore, we performed long-term studies to find answers for some of these questions.

Humoral IgG antibody plays an important role in protection against paralytic disease whereas the IgA antibody (especially secretory IgA) is critical to prevent poliovirus infection and replication at primary sites of entry. Patients with wild poliovirus (WPV) paralysis had significantly lower IgA levels than non-polio acute flaccid paralysis (AFP) [24]. Patients with paralytic poliomyelitis showed significantly lower IgG and IgA levels than non-polio AFP. Moreover, a cross-sectional survey showed that the mean serum IgG and IgA levels of children with AFP (n = 979) were significantly lower (IgG below 2 g/L and IgA below 0.07 g/L) than healthy children (n = 903) (IgG level-10.57 ± 4.53 (SD) g/L and IgA level- 1.2 ± 0.818 g/L). Moreover, two 7-month-old female children had lower IgG levels and absence of neutralizing polio antibodies [25]. Patients with low serum neutralizing antibody after immunization are re-infected with poliovirus [26], [27].

Our results show that immune response and protection varies based upon the vaccine and poliovirus serotype. Single or double dose IPV results in much lower levels of IgG1 titers specific for VP1 when compared to plant boosters. In addition, VP1-IgG1 titers drop to background level after one year in sharp contrast to plant boosters that maintains high-level antibody titers. Even when no plant booster was given (∼260 days), VP1-IgG1 titers were maintained at high levels. Most importantly, VP1-IgG1titers had direct correlation in conferring protection against Sabin 1 serotype. There was dose dependent correlation with <10% seropositivity when VP1-IgG1 antibody titers were very low, especially with a single dose IPV. Therefore, the decision to use single dose IPV to protect against serotype 2 raises a lot of new concerns.

Mucosal immunity plays an important role in containment of VDPV or WPV. Therefore, we investigated the level of serum IgA in mice immunized with IPV or plant boosters. Negligible VP1-IgA titers were observed with IPV, single or two doses in sharp contrast to steady increase in VP1-IgA levels in mice boosted with plant cell expressing VP1. High level VP1-IgA was maintained throughout 400 days in mice when boosted with plant cells. This is a very significant observation because poliovirus infection or re-infection happens through contaminated water and sewer. In developed countries with well-maintained closed sewer system, environmental surveillance may detect silent polio outbreaks, as done recently in Israel [4]. However, with open sewer system in many developing countries, generation of IgA on mucosal surface is the primary mode of protection. Failure of IPV to generate IgA again questions the decision to replace OPV with IPV. Most importantly for global eradication of polio and prevention of transmission of WPV, lack of mucosal immunity in IPV immunization is a major limitation.

Therefore, a cold-chain free and virus free polio booster vaccine made in plant cells offer an effective and timely solution, after a single dose IPV priming. Oral delivery of plant cells expressing VP1 generate high level VP1-IgG1 and VP1-IgA titers and they are maintained for > 400 days, even when no boosting was provided for several months. They also provided high level protection and seropositivity against all three poliovirus serotypes. Lyophilized plant cells can be stored indefinitely at ambient temperature without losing efficacy of vaccine antigens [17]. In spite of these results, one should explore limitations of the plant-made polio booster vaccine. It requires one priming step with IPV, because the group without such priming (group 10) did not generate neutralizing antibody. Because one IPV dose has been introduced throughout the globe, boosting with plant cells appear to be a great solution to eradicate polio and prevent VDPD or WPV transmission. However, in the long run, more efforts must be made to develop mucosal adjuvants to facilitate oral priming for universal application of plant based vaccines.

## 5 Conclusions

VP1-IgG1 titers in single or double dose IPV decreased dramatically from 29 days to 400 days, resulting in >50% reduction in seropositivity in double dose and <10% seropositivity in single dose IPV. VP1-IgA titers were negligible in IPV single or double dose. Lack of mucosal immunity will have negative impact on containment of vaccine derived wild type poliovirus or protection of silent polio outbreaks. Single dose IPV results in no or minimal amount of neutalizing antibodies against serotype 1 and 2 but results in neutralizing antibodies against serotype 3. Plant boosters with VP1 and FDA approved adjuvants (in other vaccines) maintains the same level of titers from 29 to 400 days and confers the same level of neutralizing antibodies against all three serotypes throughout the duration of this study. Lyophilized plant cells expressing VP1 can be stored without losing efficacy, eliminating cold chain. Long-term (>2 years) studies in dogs showed lack of toxicity of CTB fusion proteins [28]. Although vaccine studies in this investigation used lyophilized tobacco cells expressing VP1 in chloroplasts, high level expression has been achieved in lettuce chloroplasts and large biomass production is in progress [29], [30]. Virus-free, cold-chain free vaccine is therefore ready for further clinical development.

## Conflict of interest statement

Henry Daniell, has several patents in the field of chloroplast genetic engineering and production of biopharmaceuticals in chloroplasts. Google Scholar link is provided http://scholar.google.com/citations?user=7sow4jwAAAAJ&hl=en for full disclosure of these patents. However, he has no specific financial conflict of interest to declare.
